# Vaccination following the expanded programme on immunization schedule could help to reduce deaths in children under five hospitalized for pneumonia and severe pneumonia in a developing country

**DOI:** 10.3389/fped.2023.1054335

**Published:** 2023-03-27

**Authors:** Abu Sadat Mohammed Sayeem Bin Shahid, Ahmed Ehsanur Rahman, K. M. Shahunja, Farzana Afroze, Monira Sarmin, Sharika Nuzhat, Tahmina Alam, Fahmida Chowdhury, Mst Shahin Sultana, Mst Mahmuda Ackhter, Irin Parvin, Haimanti Saha, Shoeb Bin Islam, Lubaba Shahrin, Tahmeed Ahmed, Mohammod Jobayer Chisti

**Affiliations:** ^1^ International Centre for Diarrhoeal Disease Research, Bangladesh (Icddr,b), Dhaka, Bangladesh; ^2^ Poche Centre for Indigenous Health, University of Queensland, Brisbane, QLD, Australia; ^3^ Department of Pharmacology, Sir Salimullah Medical College, Dhaka, Bangladesh

**Keywords:** Bangladesh, children, immunization, pneumonia, hospitalization

## Abstract

**Background:**

Worldwide, pneumonia is the leading cause of mortality in children under the age of five. An expanded program on immunization (EPI) is one kind of evidence-based tool for controlling and even eradicating infectious diseases.

**Objectives:**

This study aimed to explore the impact of EPI vaccination, including BCG, DPT-Hib-Hep B, OPV, IPV, and PCV-10, among children from the age of 4 to 59 months hospitalized for pneumonia and severe pneumonia. Additionally, we evaluated the role of 10 valent pneumococcal conjugate vaccines alone on clinical outcomes in such children.

**Methods:**

In this retrospective chart review, children from the age of 4 to 59 months with WHO-defined pneumonia and severe pneumonia admitted to the Dhaka Hospital of the International Centre for Diarrheal Disease Research, Bangladesh (icddr,b) between August 2013 and December 2017 who had the information on immunization as per EPI schedule by 4 months of age were included in the analysis. A comparison was made between the children who were fully immunized (immunization with BCG, DPT-Hib-Hep B, OPV, and IPV from 2013 to 2015 and PCV-10 from 2015 to 2017) and who were not immunized (consisting of partial immunization and no immunization) during the study period.

**Results:**

A total of 4,625 children had pneumonia and severe pneumonia during the study period. Among them, 2,605 (56.3%) had received the information on immunization; 2,195 (84.3%) were fully immunized by 4 months of age according to the EPI schedule and 410 were not immunized. In the log-linear binomial regression analysis, immunization of children from 4 to 59 months of age was found to be associated with a lower risk of diarrhea (*p* = 0.033), severe pneumonia (*p* = 0.001), anemia (*p* = 0.026), and deaths (*p* = 0.035). Importantly, the risk of developing severe pneumonia (1054/1,570 [67%] vs. 202/257 [79%], *p* < 0.001) and case-fatality rate (57/1,570 [3.6%] vs. 19/257 [7.4%], *p* = 0.005) was still significantly lower among those who were immunized with PCV-10 than those who were not.

**Conclusion:**

Children immunized as per the EPI schedule were at a lower risk of diarrhea, severe pneumonia, anemia, and death, compared to unvaccinated children. In addition, PCV-10 was found to be protective against severe pneumonia and deaths in vaccinated children. The overall results underscored the importance of the continuation of immunization, scrupulously adhering to the EPI schedule to reduce the risk of morbidities and mortalities in children, especially in resource-limited settings.

## Introduction

Since the late eighties, pneumonia has been leading at the top among the infectious etiologies of global deaths of children under five (1, 2) and accounting for an estimated 12·8% of annual deaths beyond the neonatal period (3). In lower- and middle-income countries like Bangladesh, 13% of 100,000 regional deaths occur in children below five years of age (4). The majority of deaths occur in developing countries, in part, due to limited access to healthcare and public health interventions (5, 6). A substantial reduction in the past few decades of estimated pneumonia deaths (0·9 million in 2015 vs.1·7 million deaths in 1990) reflects not only economic development, improved nutrition, and reduced household crowding but also the use of pneumonia-specific interventions, such as improved case management, including empirical antibiotic treatment and effective vaccines against leading pneumonia pathogens (7, 8). Despite these advances, continued progress to reduce pneumonia mortality is constrained by the absence of vaccines against the remaining common pathogens. *Pneumococcus pneumoniae* is a major cause of childhood pneumonia and other invasive pediatric diseases, including meningitis and sepsis, accounting for approximately 14.5 million cases of invasive pneumococcal disease (IPD) and 826,000 child deaths worldwide. Among these estimated deaths, 741,000 are pneumonia deaths and 60,500 are deaths from meningitis. Bangladesh is one of the 10 countries with the highest number of IPD cases and IPD-related deaths among children under 5 years of age (9). Globally, 8 million episodes of serious disease are caused by *Haemophilus influenzae* type b (Hib) among children each year leading to an estimated one-half a million deaths (10). Both *Pneumococcus pneumoniae* and *Haemophilus influenzae* type B accounted for 5% or more of the etiological distribution of pneumonia in developing countries (11). The mortality rate from childhood pneumonia was much higher before 2,000 (12). By this period, the World Health Organization (WHO) identified several risk factors for deaths from childhood pneumonia on the basis of a good number of evidence generated from resource-limited settings and came up with a set of recommendations, including the incorporation of vaccines, appropriate antibiotics, oxygen therapy, and other routine clinical care (13) to reduce pneumonia-related deaths in children. As a result, between 2,000 and 2018, global pneumonia-related deaths among children under 5 were reduced by 54% (4).

Importantly, the introduction of *Haemophilus influenzae* type B (Hib) conjugate vaccine and pneumococcal conjugate vaccine 10 (PCV-10) in the EPI schedule by many governments in developing countries has had an enormous impact on the reduction of the pneumonia-related global burden of pneumonia caused by *Pneumococcus pneumoniae* and *Haemophilus influenzae* type B, as well as mortality in children under five years of age (14). It is also important to note that if immunization following the EPI schedule is ensured, Hib and PCV-10 as part of this schedule are simultaneously ensured (14).

EPI vaccination includes immunization against tuberculosis with Bacillus Calmette-Guerin (BCG) at birth, immunization against Diphtheria, Pertussis, Tetanus, Hepatitis B, *Haemophilus influenzae* type B with DPT-Hib-Hep B, Poliomyelitis with oral polio vaccine and inactivated polio vaccine, and *Pneumococcus pneumoniae* with PCV-10 at the 6th week, 10th week, and 14th week of birth (15). However, despite the WHO-recommended vaccines, antibiotics, oxygen therapy, and other supportive care, deaths from childhood severe pneumonia exceed 10% in many hospitals in developing countries (16, 17).

Among the 30 pneumonia-high-burden countries, Bangladesh has incorporated Hib and PCV-10 vaccines in the EPI schedule in 2009 and 2015, respectively which is considered to be on track along with Indonesia and China in order to achieve the Global Action Plan for Pneumonia and Diarrhea (GAPPD) with the goal to end pneumonia and diarrhea-related deaths by 2025 (14). Thus, Bangladesh needs to continue to keep its highly achieved 8.1% average annual rate of reduction in pneumonia-related deaths (14) to attain GAPPD by 2025. Although immunization coverage following the EPI schedule is still high in Bangladesh (18), we need to understand whether immunization has any role in the reduction of the severity of pneumonia and co-morbidities and thereby increasing the survival benefit among children from 4 to 59 months of age hospitalized for pneumonia and severe pneumonia. Thus, we sought to evaluate the impact of immunization following the EPI schedule as well as the role of PCV-10 alone on clinical outcomes in such children.

## Materials and methods

### Ethical statement

De-identified data were analyzed in this chart analysis; therefore, informed consent was not taken from the parents or guardians of the study participants. We have taken a waiver of approval by the Ethical Review Committee of the International Centre for Diarrheal Disease Research, Bangladesh (icddr,b) to use the de-identified data.

### Study site

The study was conducted in the Dhaka Hospital of icddr,b. It's an urban tertiary care hospital situated in the capital city of Bangladesh and provides both inpatient and outpatient care to diarrheal patients associated with or without co-morbidities, like pneumonia and severe pneumonia. The other descriptions of the hospital have been provided somewhere else (19).

### Study population

Children of either sex from the age of 4 to 59 months with WHO-defined pneumonia and severe pneumonia admitted into the Dhaka Hospital of icddr,b between August 2013 and December 2017 who had received the information on immunization captured from electronic medical records were enrolled in the study. Among the enrolled children, those who were fully immunized [children who received a single dose of BCG and three doses of DPT-Hib-Hep B (Diphtheria, Pertussis, Tetanus, *Haemophilus influenzae* type B, and Hepatitis-B), OPV (oral polio vaccine), and IPV (inactivated polio vaccine) during the period from 2013 to 2017 and PCV-10 (pneumococcal conjugate vaccine-10) during the period from 2015 to 2017] or not immunized (combination of partial immunization and no immunization) as per the EPI schedule by 4 months of age were selected for this analysis.

### Study design

The data were extracted from the electronic database of the Dhaka Hospital of icddr,b. We purposefully excluded children below 4 months since we needed at least 14 weeks after birth to accomplish EPI scheduled vaccination, including vaccination against *Pneumococcus pneumoniae* and *Haemophilus influenzae*. Thus, we anticipated that by 4 months of age, all the children should have received vaccination against *Pneumococcus pneumoniae* and *Haemophilus influenzae* type B. The comparison was made between children who were fully immunized as per the EPI schedule and those who were not. We didn't consider children whose vaccination records were unavailable in the electronic database for the analysis.

As Bangladesh incorporated the PCV-10 against *Pneumococcus pneumoniae* in 2015, we also compared the characteristics of children who received the PCV-10, compared to those who did not receive the same vaccine between March 2015 and December 2017.

### Measurements

We developed the case report forms which were pretested and finalized for the acquisition of the study as relevant data by the research team. The data extracted from the hospital's electronic database were routinely audited by the expert panel of the hospital's clinicians for better clarity before analysis. We evaluated the clinical and laboratory characteristics of children who were immunized, compared to those who were not (Table 1). Among the variables, diarrhea, severe acute malnutrition, hypoxemia, bacteremia, and anemia were defined following the WHO definition (13). We also used the evidence-based definition of severe sepsis (19). Finally, log-linear binomial regression analysis was done to see the independent impact of immunization.

**Table 1:** Characteristics of children hospitalized for pneumonia and severe pneumonia and had immunization following EPI schedule, compared to those who were not immunized.

Parameters	Immunized (*n* = 2195) (%)	Not immunized (*n* = 410) (%)	RR	95% CI	*p*-value
Female	747 (34)	153 (37)	1.05	0.97–1.14	0.199
Age in months (median, IQR)	9.23 (6.49, 13.23)	8.07 (5.63, 12.00)	–	–	<0.001
Presence of severe acute malnutrition on admission	911 (42)	209 (51)	0.81	0.73–0.91	<0.001
Presence of hypoxemia (SpO_2 _< 90%) on admission	611 (28)	130 (32)	0.88	0.75–1.03	0.111
Presence of severe sepsis on admission	188 (9)	50 (12)	0.70	0.52–0.94	0.019
Presence of convulsion on admission	298 (14)	57 (14)	0.98	0.75–1.27	0.859
Presence of bacteremia on admission	144 (7)	33 (8)	0.93	0.66–1.32	0.693
Presence of diarrhea on admission	1,830 (84)	368 (90)	0.93	0.89–0.97	0.002
Presence of severe pneumonia on admission	1,487 (68)	322 (79)	0.86	0.81–0.91	<0.001
Presence of anemia on admission	416 (22)	99 (29)	0.77	0.64–0.92	0.008
Duration of hospital stay (days) (median, IQR)	6.00 (4.00, 9.00)	6.00 (4.00, 9.00)	–	–	0.121
Death during hospitalization	91 (4)	34 (8)	0.50	0.34–0.73	<0.001

RR, relative risk; CI, confidence interval; IQR, inter-quartile range.

### Data analysis

We analyzed the data using SPSS for Windows (version 20.0) and STATA (version 12.0). Differences in proportion were compared by the Chi-square test. Initially, we did a bivariate analysis using a 2/2 table for the Chi-square test, and those who had significant associations between the groups by the Chi-square test like age, severe acute malnutrition, severe sepsis, diarrhea, severe pneumonia, anemia, and death were put into the log-linear binomial regression model. In the model, vaccination was the dependent variable, whereas the other significant variables in the bivariate analysis were considered independent variables. Among those variables, potential confounders like age, severe acute malnutrition, and severe sepsis were adjusted using the regression model to find out the ultimate significant risk factors having a probability of less than 0.05. The strength of the association was determined by calculating the risk ratio and their 95% confidence intervals.

## Results

A total of 4,625 children were hospitalized with pneumonia and severe pneumonia during the study period. Among them, 2,605 (56.3%) had immunization records; 2,195 (84.3%) were fully immunized by 4 months of age according to the EPI schedule and 410 were not immunized. The study found that children who were immunized more often grew to an older age and less often had severe acute malnutrition, severe sepsis, diarrhea, severe pneumonia, and anemia, compared to those who were not immunized (Table 1). Co-morbidities like hypoxemia, convulsion, and bacteremia were also comparable between the groups (Table 1). The case-fatality rate was also significantly lower among the children who were immunized than those who were not immunized (Table 1). After adjusting with potential confounders such as age, severe acute malnutrition, and severe sepsis using log-linear binomial regression analysis, immunization was found to be associated with a lower risk of diarrhea, severe pneumonia, anemia, and deaths (Table 2). Our sub-analysis found that from March 2015 to December 2017, 1,570 children received the PCV-10 and 257 did not receive the PCV-10. We also found that the risk of developing severe pneumonia (1054/1,570 [67%] vs. 202/257 [79%], *p* < 0.001) and case-fatality rate (57/1,570 [3.6%] vs. 19/257 [7.4%], *p* = 0.005) was still significantly lower among those who were immunized with PCV than those who did not receive this vaccine.

**Table 2:** Results of log-linear binomial regression analysis to understand the real impact of immunization among children from the age of 4 to 59 months hospitalized for pneumonia and severe pneumonia.

Parameters	RR	95% CI	*p*-value
Age	0.98	0.97–1.00	0.093
Severe acute malnutrition	0.97	0.79–1.20	0.783
Severe sepsis	0.98	0.71–1.38	0.914
Diarrhea	0.69	0.50–0.97	0.033
Severe pneumonia	0.60	0.44–0.80	0.001
Anemia	0.79	0.64–0.97	0.026
Death	0.67	0.46–0.97	0.035

## Discussion

Tremendous progress has been made over the last 20 years toward the development of an effective national immunization program throughout the world. The major contributor to this success is the EPI of the WHO usually implemented through UNICEF (20). It is not only associated with the reduction of disease burden but also the severity of the diseases and fatal outcomes. With the support of the GAVI Alliance (formerly known as the Global Alliance for Vaccines and Immunization), the Bangladesh government introduced the Hib and PCV-10 vaccines in the EPI schedule in 2009 and 2015, respectively, which were significant milestones for our nation. In order to understand the impact of the routine EPI vaccination by 4 months of age that includes the Hib and the PCV vaccines, we found that the vaccination was not only associated with a lower risk of morbidities but also with a lower risk of mortality, even after adjusting with potential confounders. In this study, we identified four main findings among the children from the age of 4 to 59 months who were immunized according to the EPI schedule, compared to those who were not immunized: these findings were significantly lower risk of deaths, severe pneumonia, anemia, and diarrhea.

The study's key observation was the significantly lower risk of mortality during hospitalization after immunization following the EPI schedule by 4 months of age. All the EPI vaccines either produced antibodies or activated immunologically competent cells through the stimulation of the body`s immune system and thus protected the children from vaccine-preventable diseases (21). However, all the children in our study had pneumonia with different severity and the significant reduction in deaths in these children may be attributed to the intake of Hib and PCV-10 vaccines, which were also consistent with previous observations (10, 22). Both vaccines effectively reduced the burden of pneumonia by targeting two of the most common bacterial etiologies (23). It is important to note that the PCV-10 vaccine was introduced in 2015, which was during our study period. The sub-analysis of the data during this specific period found that the children who were vaccinated with the PCV-10 also had a significantly lower risk of death, compared to those who did not receive the vaccine. This observation also bolsters the impact of PCV-10 in lowering deaths in the children in our study, which was also observed in a previous study (6, 9). PCV-10 had been documented to be safe and effective in reducing illnesses and deaths caused by *Pneumococcus pneumoniae* in numerous studies. In most high-income countries, PCV-10 was used routinely with a concomitant, and there was a substantial reduction of pneumococcal diseases, which is consistent with our study findings (24–26). Although we don`t have data on the use of empirical antibiotic therapy and effective vaccines against major pathogens causing pneumonia, we can still speculate that these might lead to a substantial reduction of mortality in our study population (7, 8).

Moreover, the heterogeneous impact of BCG in lowering deaths in children with pneumonia was also well recognized (27).

The observation of a lower risk of severe pneumonia among the immunized children, compared to the unimmunized children is also understandable. We have further identified that the children who received PCV-10 had a significantly lower risk of developing severe pneumonia, compared to those who did not receive the vaccine (21). The vaccination against pertussis might have a confounding effect in reducing pneumonia-related mortality among children who were vaccinated with PCV-10 (28, 29).

The observation of a lower risk of anemia among the immunized children, compared to the unimmunized children is really notable here. To our knowledge, this is the first study that identified the independent lower risk of anemia among immunized children. This might be the impact of an overall improved immunity potentially caused either by the production of antibodies or activation of immunologically competent cells through the stimulation of the body`s immune system among the vaccinated children (21). There was also a persistence of immunity in children who had the PCV vaccination as was observed in a previous study (30). The observation of a lower risk of diarrhea among the immunized children, compared to unimmunized children is also consistent with the previous study (31).

The main limitation of the study was its retrospective nature which potentially limited our ability to identify the association of other variables of interest. The lack of information on full vs. partial immunization was one of the limitations of our study. Another limitation was the lack of availability of 44% of information on immunization in the electronic database which led to a potential selection bias that might have evaded stronger associations. Another important limitation was that the impact of immunization in our analysis might be confounded by other WHO-recommended interventions such as antibiotics, oxygen therapy, and other routine care. The main strength of this study is the reasonably large sample from an electronic database that increases the reliability of our observation.

## Conclusion

The results of our data suggested that the children from 4 to 59 months of age who were immunized according to the EPI schedule and hospitalized for pneumonia and severe pneumonia had significantly lower case-fatality-rate, compared to those who were not immunized. The immunized children also had a lower risk of developing severe pneumonia, anemia, and diarrhea than their contemporaries. The overall results underscored the importance of increasing the national immunization coverage as high as 100%, scrupulously following the EPI schedule in order to reduce morbidities and pneumonia-related mortality in children, especially in resource-constrained settings.

## Data Availability

The raw data supporting the conclusions of this article will be made available by the authors, without undue reservation.

## References

[1] McAllister DA Liu L Shi T Chu Y Reed C Burrows J Global, regional, and national estimates of pneumonia morbidity and mortality in children younger than 5 years between 2000 and 2015: a systematic analysis. Lancet Glob Health. (2019) 7(1):e47–57. 10.1016/S2214-109X(18)30408-X 30497986 PMC6293057

[2] Perin J Mulick A Yeung D Villavicencio F Lopez G Strong KL Global, regional, and national causes of under-5 mortality in 2000–19: an updated systematic analysis with implications for the sustainable development goals. Lancet Child Adolesc Health. (2022) 6(2):106–15. 10.1016/S2352-4642(21)00311-4 34800370 PMC8786667

[3] Liu L Oza S Hogan D Chu Y Perin J Zhu J Global, regional, and national causes of under-5 mortality in 2000–15: an updated systematic analysis with implications for the sustainable development goals. Lancet. (2016) 388(10063):3027–35. 10.1016/S0140-6736(16)31593-8 27839855 PMC5161777

[4] Fighting for Breath in Bangladesh. A call to action to stop children dying from pneumonia. 3rd edn. Save the Children, UNICEF (2019).

[5] Izadnegahdar R Cohen AL Klugman KP Qazi SA. Childhood pneumonia in developing countries. Lancet Respir Med. (2013) 1(7):574–84. 10.1016/S2213-2600(13)70075-4 24461618 PMC9014772

[6] Saha S Hasan M Kim L Farrar JL Hossain B Islam M Epidemiology and risk factors for pneumonia severity and mortality in Bangladeshi children&lt; 5 years of age before 10-valent pneumococcal conjugate vaccine introduction. BMC Public Health. (2016) 16:1–12. 10.1186/s12889-016-3897-9 27927201 PMC5142317

[7] Feikin DR Flannery B Hamel MJ Stack M Hansen PM. Vaccines for children in low-and middle-income countries. Reproductive Maternal Newborn Child Health Disease Control Priorities. (2006) 2:187–205.

[8] Lee LA Franzel L Atwell J Datta SD Friberg IK Goldie SJ The estimated mortality impact of vaccinations forecast to be administered during 2011–2020 in 73 countries supported by the GAVI alliance. Vaccine. (2013) 31:B61–72. 10.1016/j.vaccine.2012.11.035 23598494

[9] O'brien KL Wolfson LJ Watt JP Henkle E Deloria-Knoll M McCall N Burden of disease caused by Streptococcus pneumoniae in children younger than 5 years: global estimates. Lancet. (2009) 374(9693):893–902. 10.1016/S0140-6736(09)61204-6 19748398

[10] Watt JP Wolfson LJ O'Brien KL Henkle E Deloria-Knoll M McCall N Burden of disease caused by Haemophilus influenzae type b in children younger than 5 years: global estimates. Lancet. (2009) 374(9693):903–11. 10.1016/S0140-6736(09)61203-4 19748399

[11] O'Brien KL Baggett HC Brooks WA Feikin DR Hammitt LL Higdon MM Causes of severe pneumonia requiring hospital admission in children without HIV infection from Africa and Asia: the PERCH multi-country case-control study. Lancet. (2019) 394(10200):757–79. 10.1016/S0140-6736(19)30721-4 31257127 PMC6727070

[12] Duke T Tamburlini G Silimperi D. Paediatric quality care G. Improving the quality of paediatric care in peripheral hospitals in developing countries. Arch Dis Child. (2003) 88(7):563–5. 10.1136/adc.88.7.563 12818896 PMC1763179

[13] Organization WH. Pocket book of hospital care for children: Guidelines for the management of common childhood illnesses. World Health Organization (2013). 24006557

[14] UNICEF. Editor UNICEF analysis based on WHO and maternal and child epidemiology estimation group interim estimates produced in September 2019, applying cause fractions for the year 2017 to united nations inter-agency group for child mortality estimation estimates for the year 2018. Convention on the Rights of the Child (2019).

[15] Sarkar PK Kumar Sarker N Doulah S Bari TIA. Expanded programme on immunization in Bangladesh: a success story. Bangladesh J Child Health. (2015) 39(2):93–8. 10.3329/bjch.v39i2.31540

[16] Chisti MJ Harris JB Carroll RW Shahunja KM Shahid A Moschovis PP Antibiotic-Resistant bacteremia in young children hospitalized with pneumonia in Bangladesh is associated with a high mortality rate. Open Forum Infect Dis. (2021) 8(7):ofab260. 10.1093/ofid/ofab260 34277885 PMC8280371

[17] Organization WH. Levels and Trends in Child Mortality: Report 2019. The World Bank. (2019).

[18] Sheikh N Sultana M Ali N Akram R Mahumud RA Asaduzzaman M Coverage, timelines, and determinants of incomplete immunization in Bangladesh. Trop Med Infect Dis. (2018) 3(3):72. 10.3390/tropicalmed3030072 30274468 PMC6160906

[19] Chisti MJ Salam MA Smith JH Ahmed T Pietroni MA Shahunja KM Bubble continuous positive airway pressure for children with severe pneumonia and hypoxaemia in Bangladesh: an open, randomised controlled trial. Lancet. (2015) 386(9998):1057–65. 10.1016/S0140-6736(15)60249-5 26296950

[20] Muaz SSA. Introducing conjugate pneumococcal vaccine at EPI schedule in Bangladesh-impact on sustainable development goal. Bangladesh J Child Health. (2015) 39(2):65–8. 10.3329/bjch.v39i2.31534

[21] Berical AC Harris D Dela Cruz CS Possick JD. Pneumococcal vaccination strategies. An update and perspective. Ann Am Thorac Soc. (2016) 13(6):933–44. 10.1513/AnnalsATS.201511-778FR 27088424 PMC5461988

[22] Wahl B O'Brien KL Greenbaum A Majumder A Liu L Chu Y Burden of Streptococcus pneumoniae and Haemophilus influenzae type b disease in children in the era of conjugate vaccines: global, regional, and national estimates for 2000–15. Lancet Glob Health. (2018) 6(7):e744–e57. 10.1016/S2214-109X(18)30247-X 29903376 PMC6005122

[23] Madhi SA Levine OS Hajjeh R Mansoor OD Cherian T. Vaccines to prevent pneumonia and improve child survival. Bull World Health Organ. (2008) 86(5):365–72. 10.2471/BLT.07.044503 18545739 PMC2647441

[24] Constenla DO. Post-introduction economic evaluation of pneumococcal conjugate vaccination in Ecuador, Honduras, and Paraguay. Revista Panamericana de Salud Publica. (2015) 38:388–95. 26837524

[25] Diaz J Terrazas S Bierrenbach AL Toscano CM Alencar GP Alvarez A Effectiveness of the 10-valent pneumococcal conjugate vaccine (PCV-10) in children in Chile: a nested case-control study using nationwide pneumonia morbidity and mortality surveillance data. PLoS One. (2016) 11(4):e0153141. 10.1371/journal.pone.0153141 27058873 PMC4825990

[26] Von Mollendorf C Tempia S Von Gottberg A Meiring S Quan V Feldman C Estimated severe pneumococcal disease cases and deaths before and after pneumococcal conjugate vaccine introduction in children younger than 5 years of age in South Africa. PloS One. (2017) 12(7):e0179905. 10.1371/journal.pone.0179905 28671978 PMC5495214

[27] Roy P Vekemans J Clark A Sanderson C Harris RC White RG. Potential effect of age of BCG vaccination on global paediatric tuberculosis mortality: a modelling study. Lancet Glob Health. (2019) 7(12):e1655–e63. 10.1016/S2214-109X(19)30444-9 31708146 PMC7024998

[28] Simonsen L Taylor RJ Schuck-Paim C Lustig R Haber M Klugman KP. Effect of 13-valent pneumococcal conjugate vaccine on admissions to hospital 2 years after its introduction in the USA: a time series analysis. Lancet Respir Med. (2014) 2(5):387–94. 10.1016/S2213-2600(14)70032-3 24815804

[29] Barger-Kamate B Deloria Knoll M Kagucia EW Prosperi C Baggett HC Brooks WA Pertussis-associated pneumonia in infants and children from low-and middle-income countries participating in the PERCH study. Clin Infect Dis. (2016) 63(suppl_4):S187–S96. 10.1093/cid/ciw546 27838672 PMC5106621

[30] Janapatla RP Hsu M-H Chen C-L Wei S-H Yu M-J Su L-H Persistence of immunity in children immunised with 13-valent pneumococcal conjugate vaccine and impact on nasopharyngeal carriage: a cross-sectional study. Thorax. (2020) 75(8):689–92. 10.1136/thoraxjnl-2019-213878 32444435 PMC7402562

[31] Chang AY Riumallo-Herl C Salomon JA Resch SC Brenzel L Verguet S. Estimating the distribution of morbidity and mortality of childhood diarrhea, measles, and pneumonia by wealth group in low-and middle-income countries. BMC Med. (2018) 16(1):1–13. 10.1186/s12916-017-0981-7 PMC6030776 29970074

